# WOMAN-PharmacoTXA trial: Study protocol for a randomised controlled trial to assess the pharmacokinetics and pharmacodynamics of intramuscular, intravenous and oral administration of tranexamic acid in women giving birth by caesarean section

**DOI:** 10.12688/wellcomeopenres.16884.1

**Published:** 2021-06-16

**Authors:** Monica Arribas, Ian Roberts, Rizwana Chaudhri, Amber Geer, Danielle Prowse, Mwansa Ketty Lubeya, Aasia Kayani, Kiran Javaid, Stanislas Grassin-Delyle, Haleema Shakur-Still

**Affiliations:** 1 Clinical Trials Unit, London School of Hygiene & Tropical Medicine, London, WC1E 7HT, UK; 2 Global Institute of Human Development, Shifa Tameer-e-Millat University, Islamabad, H-8, Pakistan; 3 Women and Newborn Hospital, University Teaching Hospital, Nationalist Road, Lusaka, PB RW1X, Zambia; 4 Department of Obstetrics and Gynaecology, The University of Zambia-School of Medicine, Lusaka, Zambia; 5 Département de Biotechnologie de la Santé, Université Paris-Saclay, UVSQ, Inserm, Infection et inflammation, Montigny le Bretonneux, France; 6 Département des Maladies des Voies Respiratoires, Hôpital Foch, Suresnes, France

**Keywords:** Antifibrinolytic, clinical trial, Tranexamic acid, PPH, intramuscular, oral, Pharmacokinetics, Pharmacodynamics, caesarean section

## Abstract

**Background:** Intravenous tranexamic acid (TXA) within 3 hours of birth significantly reduces death due to bleeding in women with postpartum haemorrhage (PPH). Most PPH deaths occur in the first hours after giving birth and treatment delay decreases survival.  One barrier to rapid TXA treatment is the need for intravenous injection. Intramuscular injection and oral solution of TXA would be easier and faster to administer and would require less training. However, the pharmacokinetics (PK), pharmacodynamics and safety of TXA administered by different routes in pregnant women have not been established. The main aim of this study is to ascertain whether IM and oral solution of TXA will be absorbed at levels sufficient to inhibit fibrinolysis in pregnant women.

**Methods:** WOMAN-PharmacoTXA is a prospective, randomised, open label trial to be conducted in Zambia and Pakistan.  Adult women undergoing caesarean section with at least one risk factor for PPH will be included.  Women will be randomised to receive one of the following about 1 hour prior to caesarean section: 1-gram TXA IV, 1-gram TXA IM, 4-grams TXA oral solution or no TXA. Randomisation will continue until 120 participants with at least six post randomisation PK samples are included. TXA concentration in maternal blood samples will be measured at baseline and at different time points during 24 hours after receipt of intervention. Blood TXA concentration will be measured from the umbilical cord and neonate.

The primary endpoint is maternal blood TXA concentrations over time. Secondary outcomes include umbilical cord and neonate TXA concentration D-dimer concentration, blood loss and clinical diagnosis of PPH, injection site reactions and maternal and neonate adverse events.

**Discussion:** The WOMAN-PharmacoTXA trial will provide important data on pharmacokinetics, pharmacodynamics and safety of TXA after IV, intramuscular and oral administration in women giving birth by caesarean section.

**Trial registration:** ClincalTrials.gov, NCT04274335 (18/02/2020).

## Abbreviations

BIC: Bayesian Information Criterion; CHMP: Committee for Medicinal Products for Human Use; CI: Chief Investigator; CI: Confidence Interval; Cmax: Maximum Concentration; COV: Covariate; CS: Caesarean Section; CRF: Case Report Form; CTU: Clinical Trials Unit; DMC: Data Monitoring Committee; EMA: European Medical Agency; FBC: Full Blood Count; g: Gram; GCP: Good Clinical Practice; h: Hour; ICH-GCP: International Conference on Harmonisation-Good Clinical Practice; IM: Intramuscular; IMP: Investigational Medicinal Product; IV: Intravenous; L: Litre; LMICS: Low and Middle-Income Countries; LSHTM: London School of Hygiene & Tropical Medicine; MCMC: Markov Chain Monte Carlo; MedDRA: Medical Dictionary for Regulatory Activities; min: Minute; mg: Milligram; mL: Millilitre; µL: Microlitre; OR: Odds Ratio; PD: Pharmacodynamics; PI: Principal Investigator; PK: Pharmacokinetics; PPH: Post-Partum Haemorrhage; PWR: Power; RCT: Randomised Control Trial; RR: Relative Risk; SAEM: Stochastic Approximation Expectation Maximization; SUSAR: Suspected Unexpected Serious Adverse Reaction; TXA: Tranexamic Acid.

## Background

Postpartum haemorrhage (PPH) is a leading cause of maternal mortality and morbidity. About 14 million women suffer a PPH every year after childbirth and about 100,000 die if bleeding is not controlled ^1^. Most PPH deaths occur in low- and middle-income countries (LMICs) ^2– 5^. Of those who survive, many suffer severe morbidity and need major interventions to control bleeding, including exploratory laparotomy, brace sutures and hysterectomy.

Tranexamic acid (TXA) is an antifibrinolytic drug. It binds to the lysine receptors on plasminogen blocking the conversion from plasminogen to the proteolytic enzyme plasmin, thereby preventing fibrin degradation and stabilising blood clots ^6, 7^. Intravenous TXA reduces blood loss in surgery and increases survival in traumatic bleeding and PPH. The WOMAN trial showed that giving intravenous TXA reduced the risk of death from PPH ^8^. TXA treatment, as early as possible and no later than 3 hours, reduced PPH deaths by one third and the need for laparotomy to control bleeding by over one third.

Most deaths from PPH occur within the first hours after giving birth. The survival benefit of TXA reduces by 10% with every 15 minutes of treatment delay, and after 3 hours there is no benefit ^9^. The need for intravenous administration of TXA is one of the main barriers for rapid treatment. Currently, TXA for PPH can only be given where trained healthcare professionals are available to give the drug intravenously. However, in many LMIC countries, a large number of women give birth at home with no access to trained personnel. Additionally, many nurses and midwives who care for women with PPH are not trained to give intravenous drugs.

 Alternatives routes to IV TXA would make administration easier and faster and would require less training. TXA is available for oral administration (tablet and solution) and has the potential to be given by intramuscular injection.

However, there has been little research into different routes of administration and the pharmacokinetic (PK) properties of TXA in pregnant women have not yet been established.

Studies in healthy volunteers show that therapeutic plasma TXA levels (plasma TXA >10 mg/L) ^10^ are reached rapidly (within 30 min) after IM injection ^11– 13^. PK modelling of the oral administration of 4 g of TXA resulted in a plasma concentration of 10 mg/L within 15 min (data on file). If absorption was similarly rapid in pregnant women, this would strongly suggest the IM and oral routes as potential alternatives to IV use.

Recently, the Trauma-INTACT trial (ClinicalTrials.gov: NCT03875937) assessed the PK of TXA administered intramuscularly in bleeding trauma patients ^14^. The study found that the time to reach therapeutic concentrations of 10 mg/L after intramuscular injection of 1 g TXA was within 15 min. Additionally, it was well tolerated with only mild injection site reactions and no serious adverse events reported. The PharmacoTXA trial (ClinicalTrials.gov: NCT03777488) has assessed the PK of TXA administered intramuscularly or orally in healthy volunteers. An ongoing RCT called TRACES (TRAnexamic Acid to Reduce Blood Loss in Hemorrhagic CESarean Delivery) aims to assess the PK and pharmacodynamics (PD) effects of two doses of TXA (0.5 and 1 g) injected intravenously in women with an ongoing PPH after delivery ^15^.

Physiologic changes induced by pregnancy and childbirth ^16^ may alter the PK and PD properties of medicines, in particular due to the increase in renal elimination clearance and volume of distribution.

PPH can occur without warning in most women. However, some risk factors such as multiple pregnancy, macrosomia or previous PPH, are known to be associated with an increased risk of PPH ^17, 18^.

In this study we will assess the PK, PD, safety and efficacy of TXA administered by IV, IM or oral routes in women giving birth by caesarean section (CS) with at least one risk factor for PPH. Blood samples will be taken from the women immediately before the administration of TXA (1 hour before CS) and at different time points during 24 hours after receipt of intervention. Blood samples from the umbilical cord after clamping and from newborns (obtained from the sample taken for routine heel prick test) will be taken to measure the amount of TXA transferred from the mother to the neonate via the placenta. The efficacy of the different routes of TXA administration in preventing blood loss, reducing fibrinolytic activity, and the safety of the interventions will be evaluated.

## Rationale

### Hypothesis

We hypothesise that IM and oral solution of TXA will be well absorbed in pregnant women. Based on PK modelling of data available in the literature, we predict that a 1 g IM injection or 4 g oral solution administration of TXA will provide therapeutic TXA levels ≥10 mg/L in plasma ^10^ within about 30 minutes. 

### Description of trial population

Recruitment will continue until 120 adult women who are undergoing CS with at least one risk factor for PPH and who complete the trial are included. 30 women will receive 1 gram of TXA by IV injection, 30 women will receive 1 gram of TXA by IM injection, 30 women will receive 4 grams of TXA orally, and 30 women will receive no TXA about 1 hour prior to the CS. Additionally, women will receive routine interventions for active management of 3 ^rd^ stage of labour and if they develop PPH, all standard interventions should be given. There are no restrictions on treatment of co-morbidities.

### Description of investigational product and justification of the dosage, route of administration, administration schedule and treatment duration

In this study, women will be randomised to four groups:

1. **IV TXA GROUP:** 30 women will receive 1 g of TXA by IV route. IV TXA treatment rapidly achieves the plasma levels of TXA needed to inhibit fibrinolysis and prevent excessive bleeding. Pharmacological research has shown that an IV dose of 1 g TXA maintains therapeutic plasma levels for around 3 hours, the period when the risk of bleeding is greatest ^12, 19^. However, the TXA PK and PD in pregnant women has not been described so far.
2. **IM TXA GROUP:** 30 women will receive 1 g of TXA by IM route. Studies of IM TXA in healthy volunteers report good absorption and no adverse effects ^11, 12^. The dose will be given as two 5 mL (0.5 g each) injections into the thigh (rectus femoris or vastus lateralis) or buttocks (gluteal muscles) muscles, depending on a clinical assessment of muscle mass ^12^. The 1 g dose (10 mL) is divided to reduce the volume injected (5 mL is considered the upper limit) ^20^. The injections will be given using the most appropriate needle size for IM administration from the sites stock (1" between 19 - 25 gauge and from 1 ½ inches up to 3" for large adults) using the Z-track method to seal the medication in the muscle ^20^.
3. **TXA SOLUTION ORALLY:** 30 women will receive 4 g of TXA oral solution. The dose was selected because of the 50% oral bioavailability and because previous and preliminary PK data show that the absorption through the oral route is much slower than through the IV or IM routes, with much lower maximal concentration (Cmax) ^12, 13, 19, 21^. Therefore, higher doses are needed to reach therapeutic levels, according to simulations. The oral route (tablets or solutions) is well characterised ^22– 24^, but oral TXA PK and PD in pregnant women have not been described so far.
4. **NO TXA CONTROL:** 30 women will receive no TXA and will be the control group for PD, blood loss and safety data.

### Safety of TXA and potential benefits and risks for study participants

TXA has been in clinical use for decades and has a good safety profile. TXA reduces the risk of death due to bleeding in women with PPH and in patients with traumatic bleeding, without an increase in adverse events. The WOMAN trial recruited 20,060 women with PPH and showed that TXA reduces the death due to bleeding (RR=0.81, 95% CI 0.65 to 1.00), especially when administered within three hours of giving birth (RR=0.69, 95% CI 0.52 to 0.91) ^8^. The CRASH-2 trial randomised 20,211 patients with trauma and showed that TXA reduces deaths due to bleeding when given soon after injury ^25, 26^. The combined evidence from 40,138 patients recruited in the WOMAN and CRASH-2 trials indicates that TXA significantly increases survival from bleeding (odds ratio [OR]=1·20, 95% CI 1·08–1·33; p=0·001), the effect increases to 70% when the treatment is immediate after injury/childbirth (OR=1·72, 95% CI 1·42–2·10; p<0·0001) ^9^. In surgery, intravenous TXA reduces blood loss (RR=0.66, 95% CI 0.65 to 0.67) and the need for transfusion (RR=0.62, 95% CI 0.58 to 0.68) by about a third ^27, 28^.

The most common potential side-effects reported by manufacturers to be associated with use of TXA according to frequency are diarrhoea, vomiting and nausea ^29, 30^.

Most of the TXA is excreted in the urine unchanged within 12 hours of administration. To prevent accumulation in cases of renal impairment, women with known renal insufficiency are excluded from participating in this study. In addition, because a single dose of 1 g (IV or IM) or 4 g (oral) is used in the WOMAN-PharmacoTXA trial, there will be no risk of accumulation.

The absolute risk of thromboembolism amongst pregnant and postpartum women is estimated to be around 2 to 3 per 1,000 woman-years. Even with a low risk, the incidence of thromboembolic events is four times higher amongst pregnant and post-partum women than in non-pregnant women of the same age ^31^. There was no increase in the risk of venous thrombosis in the participants treated with TXA in the WOMAN trial. Given that severe bleeding may lead to vascular occlusive events it is possible that, by reducing bleeding, TXA also reduces the risk of thrombosis ^32^.

TXA passes into breast milk in very low concentrations, approximately one hundredth of the concentration in maternal blood ^33– 35^. One study reported that growth and development parameters were similar in children exposed to TXA through breastmilk compared to unexposed children, with no long-term adverse events ^33^. No adverse events in breastfed babies were found in the WOMAN trial ^8^.

TXA administered to pregnant women crosses the placenta and TXA concentration in the umbilical cord blood is similar to the concentration in the maternal blood ^36, 37^. The half-life of TXA in serum is 1–2 hours ^38^, which would result in the elimination of TXA from the body within a few hours. Information is limited on the potential adverse effects on neonates of TXA administered to women before CS.

One study reported that no side effects were observed in new born babies from 12 healthy mothers who received TXA 10–20 min before CS delivery ^36^. In three trials, 1 g of TXA was administered 10–20 minutes before CS, and no adverse events to mothers and neonates were reported in two of them ^34^. One of the three studies reported that some mild and transient side effects occurred, but did not specify what kind of effects, how many participants were affected, and if any of these effects affected the babies ^39^. A systematic review on the prophylactic use of TXA in cases of elective CS or abdominal myomectomy included 16 trials with a total of 2,949 patients (2,789 underwent CS) ^40^. Only one of the eligible studies reported thromboembolic events that occurred in two patients in the TXA group, and two in the control group. This review did not report any side effects of prophylactic TXA on neonates. Similarly, a systematic review on the prophylactic use of TXA in women undergoing CS included 21 trials with a total of 3,852 patients ^41^. Also this review did not report any adverse events in the mothers, except for the four cases of thromboembolic events reported in the same trial included in the previous review. A review on guidelines and relevant data on the use of TXA in PPH prophylaxis for CS and vaginal delivery reported that the only evidence of possible adverse events for the mother concerned poor renal outcome in a case series study with 18 patients with renal cortical necrosis in PPH where a TXA maintenance dose was used. The case series study reported that patients that did not recover normal renal function had a TXA maintenance dose of 0.5–1 g/h for a longer time (7.1 ± 4.8 h) compared to patients who recovered partial renal function (2.9 ± 2.4 h) ^42, 43^. This review confirmed that there are no reports of adverse neonatal outcomes associated with the administration of TXA shortly before giving birth to prevent PPH ^42^. A systematic review on the effectiveness and safety of the use of TXA to prevent PPH that included 25 studies with a total of 4,747 participants concluded that there was no increased risk of deep vein thrombosis and an increased risk of minor transient events, i.e. nausea, vomiting, headache or dizziness ^44^. The included studies that evaluated the safety of TXA on neonates reported that no adverse events occurred, and the Apgar score that assesses the baby’s health immediately after birth showed no difference between babies in TXA-treated and control groups ^44^.

No mutagenic activity of TXA has been detected *in vitro* and *in vivo* test systems ^45^. No foetal abnormalities were identified in early dysmorphology and reproductive studies in animals ^45– 47^.

As the TXA will be delivered at the end of the 3rd trimester of pregnancy when the foetal development is complete, the trial treatment will not have any impact on foetal development.

The foreseeable risks related to the IM route of administration may include pain, redness and bruising at the injection site and a rare risk of infection at the injection site.

The study also involves additional blood samples from all participants. We will take the smallest possible volume of blood that will allow us to carry out the analysis. The total volume of blood taken from the participating women (maximum of 35 mL over 24 hours) should have no clinical impact on the participant. One blood sample (2 × 10 µl) will be taken from the neonate at the time of the routine heel prick test.

We will routinely assess all women for nausea, vomiting, diarrhoea, thrombotic events, seizures and injection site reactions as secondary outcomes. Additionally, all neonates will be routinely assessed for birth complications and adverse events.

## Objectives and outcome measures/endpoints

### Primary objective

To assess the population PK of IV, IM and oral TXA solution in pregnant women.

### Secondary objectives

• To assess the effect of the three routes of TXA administration on D-dimer concentration in blood samples.
• To assess the concentration of TXA that crosses the placenta into the neonate via the three routes of administration.
• To assess clearance of any TXA in neonate.
• To assess the safety of the three routes of TXA administration.
• To assess the effect of the three routes of TXA administration on postpartum bleeding.

### Primary endpoint

Blood TXA concentrations over time in pregnant women.

### Secondary endpoints

• Blood concentrations of D-dimer over time
• TXA concentration in umbilical cord and neonate after birth
• Local reactions at IM injection sites
• Adverse events (maternal and neonate)
• Neonate status Apgar score
• Measured blood loss from start of CS to 2 hours after
• Clinical diagnosis of PPH

## Trial design

A prospective, randomised, open label study to be conducted in obstetric units in Pakistan and Zambia. Potential eligible participants will undergo CS. Consent will be obtained as per section “Methods for informing and obtaining consent”.

## Trial setting

Participants will be recruited from obstetric units in Pakistan and Zambia. Recruitment, treatment and follow-up will be conducted at the recruiting obstetric units. Names of participating sites will be listed on the trial website.

## Participant eligibility criteria

### Inclusion criteria

• Women admitted to hospital giving birth by CS
• History of at least one risk factor for PPH
• Adult (≥18 years old)

### Exclusion criteria

• Women giving birth vaginally
• Women with a known allergy to TXA or its excipients
• Women with current antepartum haemorrhage
• Women known to have received TXA within 48 hours prior to randomisation
• Women with known renal impairment
• Women with any known blood clotting disorder

## Trial procedures

### Summary of trial procedures

**Figure 1.**
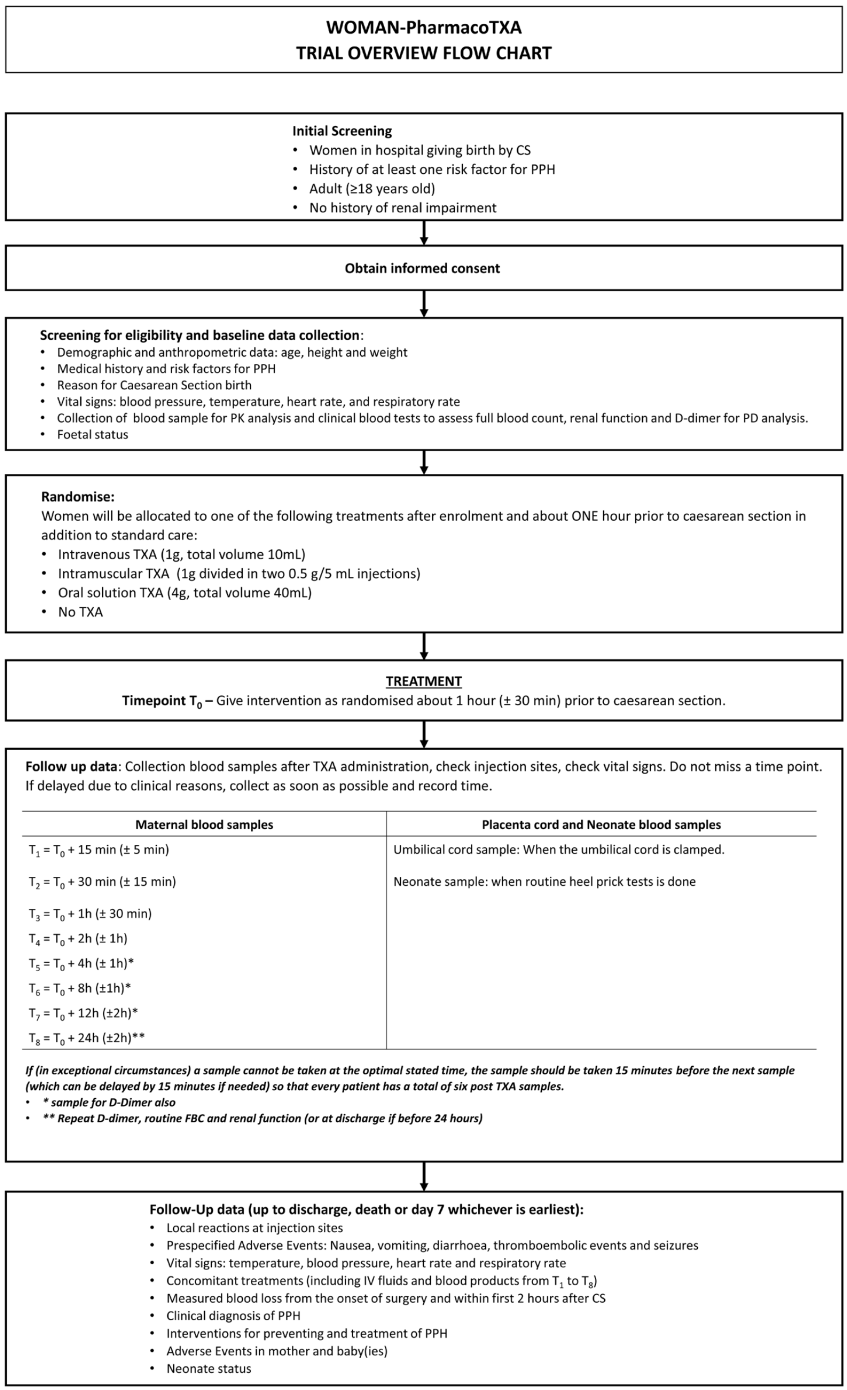
Trial overview.

**Figure 2.**
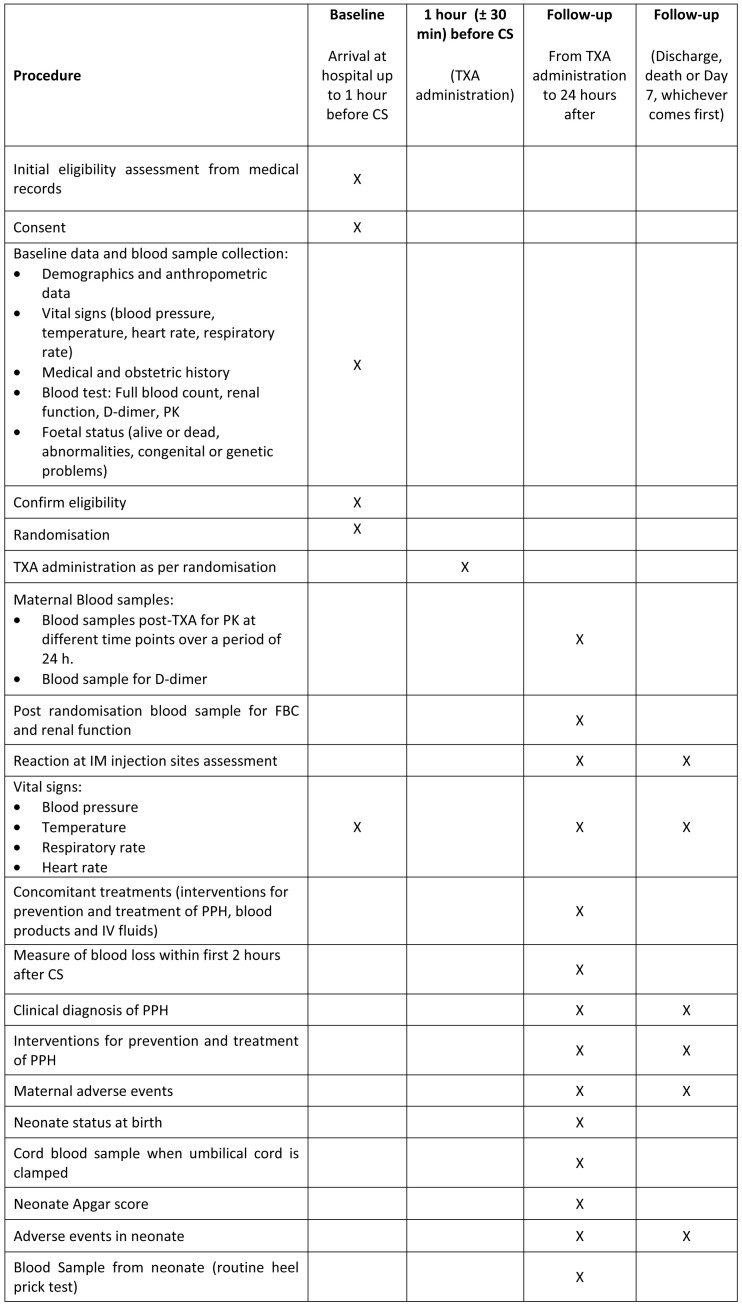
Summary of trial procedures.

The trial overview flow-chart is shown in Figure 1 the summary of trial procedures is shown in Figure 2. The protocol has been prepared according to the Standard Protocol Items: Recommendations for Interventional Trials (SPIRIT) 2013 guidelines ^48^ (Additional files 1 and 2, *Extended data*) ^49^. The complete copy of the protocol can be found in Additional file 3 (see *Extended data*) ^49^.

### Screening of potential participants

Potential eligibility will be assessed by a clinician at the participating obstetric clinic when the woman is admitted for a CS.

### Methods for informing and obtaining consent

***Information giving.*** Women who are admitted for a CS with one risk factor for PPH will be given oral and written information and fully informed consent will be obtained by the clinician. If she is unable to read or write, the information sheet will be read to her, and she will mark the consent form with a cross or thumbprint. In this case, an impartial witness must be present throughout the procedure and provide a signature confirming the mark.

The clinician will explain the trial to the woman verbally in a language that she understands and provide a written participant information sheet (Additional file 4, *Extended data*) ^49^. In brief, it will be explained that she will receive the usual care given to women having a CS at the hospital. The clinician will explain that if she accepts to participate, because she is at risk of increased post-partum bleeding she will receive a drug called tranexamic acid into a vein, or a muscle, or to swallow it, or she may receive no trial drug. We need to study this because injecting TXA into a muscle or swallowing it are easier than injecting it into a vein.

This would mean that the treatment could help many more women giving birth who are at a high risk of bleeding. The clinician will explain to the woman the expected risks of the treatment to herself and the baby. If the woman objects to inclusion, her views will be respected. The clinician will explain to the woman that she has the right to withdraw from the trial at any time, without the need to justify the reason and without any consequences on the care provided to her and her baby. An overview of the consent procedure is provided in Additional file 5 (see *Extended data*) ^49^.

***Documenting consent process.*** In all cases, the clinician obtaining consent should record in the participant’s medical notes the method used for obtaining a participant’s consent. The clinician should retain the original signed and dated consent form, a copy should be given to the woman.

### Baseline data collection

Baseline parameters will be used to build a PK model that describes the TXA concentration in the blood over time following TXA administration. 

The following baseline data will be recorded:

• Demographic (age) and anthropometric data (height and weight [body mass index and body surface area using the Du Bois formula will be derived]). Information on these parameters can be collected at baseline
• Medical and pregnancy history
• PPH risk factor(s)
• CS planned date and time
• Reason for CS
• Vital signs including blood pressure, temperature, heart rate, and respiratory rate
• Full blood count
• Blood test to assess renal function (urea, creatinine, estimated glomerular filtration rate)
• Any foetal abnormalities
• Any foetal distress

### Randomisation

After collecting baseline data, enrolled participants will be randomised into one of the four groups described in “Description of investigational product and justification of the dosage, route of administration, administration schedule and treatment duration”. The sequence for each dosing cohort will be created using computer-generated random numbers, using blocking to ensure the required balance in the allocation of participants to treatment arms. The allocation ratio will be 1:1:1:1. 

An IT coding expert supported by a statistician who are not involved in the conduct of the trial will prepare the randomisation codes. Eligibility will be confirmed and randomisation done via an online database. Once the code is generated, the trial intervention will be prepared and administered by a trained trial team member. Participants and all trial staff will be blind to treatment allocation until randomisation is completed.

### TXA administration and timing of biological samples

The time of the TXA administration is T _0_. One blood sample will be taken for PK prior to drug administration.

**Figure 3.**
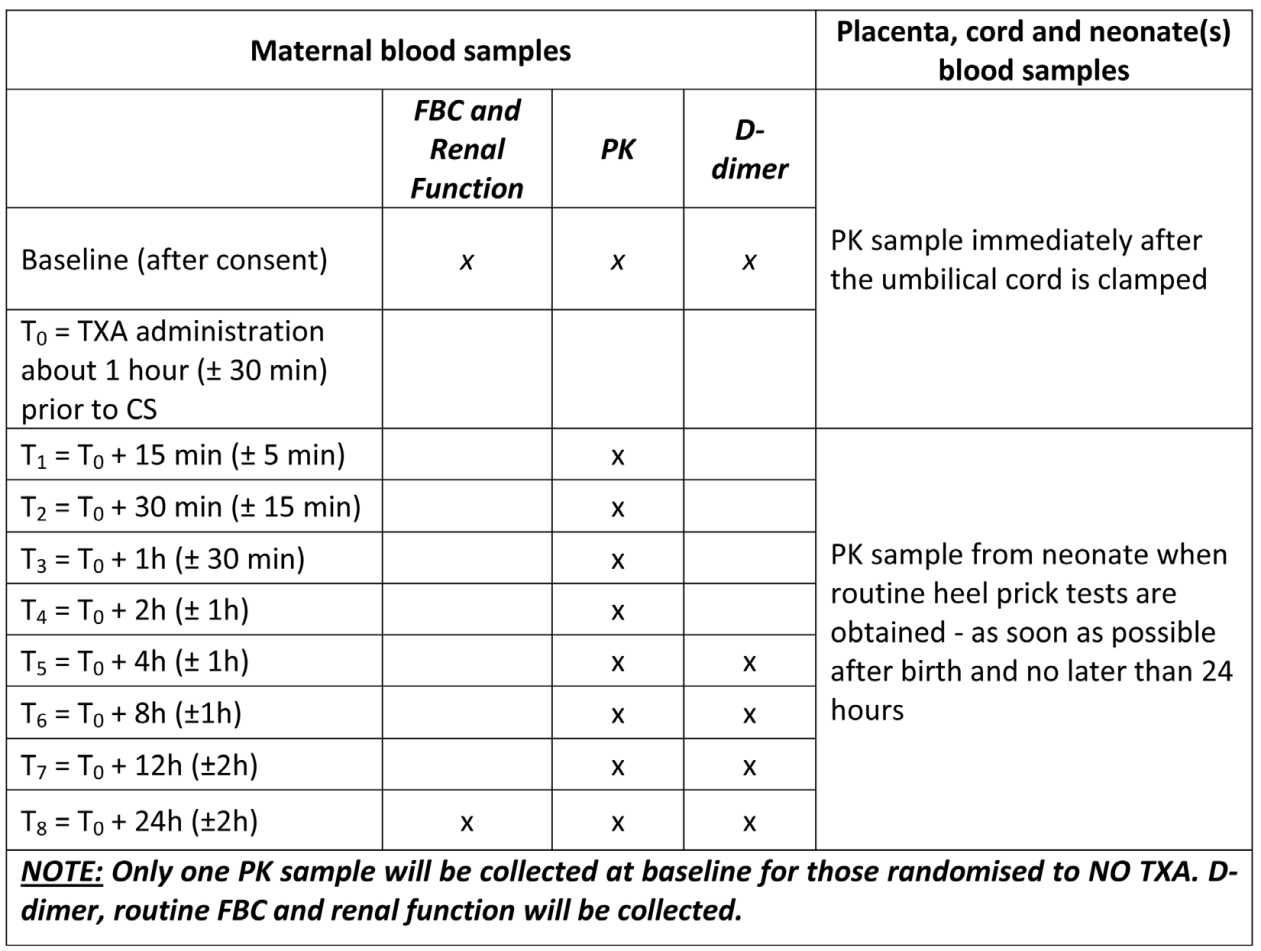
Timing of biological samples.

Post TXA administration blood samples will be taken at different time points during 24 h as per the schedules in Figure 3. We understand that the care of the participant takes priority over the sampling schema and that sample times will inevitably differ from those indicated. If it is not possible to obtain a sample at the scheduled time, the sample should be collected as soon as feasible with the exact time of collection recorded. Reasons for any delay will be recorded. Where there are less than six evaluable PK samples obtained after TXA administration from a participant, this participant will be replaced in the study.

*Pharmacokinetics*: Finger prick blood samples (2 × 10 µL per sample) for TXA quantitation will be taken as per Figure 3.

*Pharmacodynamics*: Blood samples (3 mL of venous blood in tubes containing 3.2% sodium citrate) for D-dimer quantitation will be taken as per Figure 3.

### Collection and storage of biological samples

Only one blood sample for PK analysis will be collected at baseline for women who are randomised to receiving no TXA. Samples for D-dimer, full blood count (FBC) and renal function will be collected.

Maternal blood will be taken from a cannula to avoid multiple venepuncture as follows:

Two samples of 3mL each will be taken for FBC and two samples of 3mL each for renal function. Blood samples will be collected in purple top EDTA tubes for FBC and yellow top tubes with separation gel and clot activator for renal function.

Five samples for D-dimer of 3 mL for each sample of blood to be taken in collection blue top tubes containing 3.2% sodium citrate. Blood samples for D-dimer, FBC, and renal function will be collected from the hospitals and analysed by a local central laboratory. The laboratory will be asked to store the sample used for D-dimer analysis until the end of trial to allow for repeat tests if needed. In the event of failure of any of the PK samples, this will be used as a back-up and will be destroyed at the end of the trial.

Each maternal PK sample require about 20 µL of blood taken using a Mitra ^®^ cartridge from a finger prick (2 × 10 µL samples taken on the 2 cartridges of the same device to have one primary and one confirmation sample if needed). Sites will be provided with written procedures on how to obtain the blood sample using the Mitra ^®^ cartridge. Cartridges will be labelled with the date and time the sample was taken and with the participant Study Identification number.

The total volume of blood taken (less than 35 mL) should have no clinical impact on the participant.

To obtain the umbilical PK blood sample, once the umbilical cord is clamped, it is wiped with antiseptic and a syringe and needle is inserted into the vein in the umbilical cord to withdraw a small amount of blood. PK samples from the umbilical cord and neonates will also be taken using the Mitra ^®^ cartridge system as described above.

A blood sample from the neonate (2×10 µL) will be taken using the Mitra ^®^ cartridge at the time of the routine clinical heel prick test to avoid additional heel pricks.

PK samples will be placed into suitable sealed biological sample shipping bags and sent weekly to Dr Grassin-Delyle’s laboratory at UFR Simone Veil - Santé, University Versailles Saint Quentin (2 avenue de la source de la Bièvre, 78180 Montigny le Bretonneux, France) for PK analysis. No participant identifiable data will be transferred to the laboratory. Once all analyses for this trial have been completed, all blood samples will be destroyed.

### Follow-up assessments

The following parameters will be assessed and recorded during the follow-up time of 7 days or until discharge, whichever is earlier:

**Maternal:**

**• Blood lost:** Blood lost from incision to 2 hours after the CS will be estimated. The amount of blood in sponges and drapes used in surgery and blood loss from suctioning (excluding amniotic fluid) will be estimated. At the end of surgery, a calibrated obstetric drape will be used for 2 hours. 

**• Reaction at site of IM injections**: Each IM injection site will be inspected for local reactions at the same time as PK blood sampling and then daily thereafter (for 7 days or until prior discharge).

**• Vital signs**: Participants will have their blood pressure, temperature, heart rate and respiratory rate recorded at the time of each PK blood sampling and daily until discharge or day 7.

**• Treatments:** Data on treatments likely to influence PK levels of TXA (blood product transfusion and IV fluids administration, TXA or other antifibrinolytics) will be collected from the time of TXA administration up to the time of the last PK sample.

**• Adverse events:** As described in section “Pharmacovigilance”, will be recorded up to 7 days.

**• Clinical diagnosis of PPH:** Clinical diagnosis of PPH up to 24 hours after birth (total blood loss of >1000 mL, or any blood loss sufficient to cause haemodynamic instability or requires further treatment).

**Neonate:**

**• Apgar score** recorded 1 and 5 minutes after birth. This is a standard clinical test given to all newborns. This test checks a baby's heart rate, muscle tone, and other signs to see if extra medical care or emergency care is needed.

**• Adverse events:** As described in section “Pharmacovigilance”, will be recorded up to 7 days.

### End of trial for participants

The trial ends at discharge, death or at 7 days, whichever occurs first.

### Distinction between standard care and research

The only departure from standard care is the TXA given about 1 hour prior to CS and extra blood sampling. Additionally, we will regularly inspect the IM injection sites.

### Blood sample analysis

***TXA quantitation.*** TXA will be measured with liquid chromatography coupled to mass spectrometry according to an analytical method validated following the European Medical Agency (EMA) guideline on bioanalytical method validation (EMEA/CHMP/EWP/192217/2009Rev.1 Corr.2) ^50^. The method is linear in the range 0.1–1000.0 µg/mL, accuracy is between 85.9 and 110.8% and precision <8.2%.

***D-dimer quantitation.*** Blood for D-dimer assays will be assayed at a central laboratory. A citrate-containing tube will be properly filled and mixed via inversion. It will be transported to the laboratory and analysed within 3 hours. Quantification will be done by immunoassay.

### Premature exit of trial participant

Participants may exit the study at any time and for any reason. A previously given consent can be withdrawn. The investigator can withdraw a participant from the trial for any safety reason or if it is in the participant's best interest.

If a participant exits the trial prematurely or withdraws consent or refuses consent for continuation, data collected up to time of premature exit will be used. Participants who withdraw from the trial with less than six post treatment PK samples will be replaced to ensure the four intervention groups remain balanced.

***Monitoring participants after the premature termination of treatment.*** In case of an adverse event to the participant or her baby, the investigator will complete an adverse event report and monitor the event until the end of her participation in the research or until it has resolved or reached a stable state.

***Procedure for replacing participants.*** If consent procedures are complete but the TXA dose is not given in full, the participant will be replaced.

When oral TXA is given and the participant vomits within the first hour of receiving the intervention, this participant will be replaced in the study. However, data collection for the participant will continue to the trial end.

Where the CS is delayed and takes place more than 2 hours after the administration of the intervention, the participant will be replaced in the study. However, data collection for the participant will continue to the trial end.

If a participant receives the TXA dose but there are less than six post treatment evaluable PK samples obtained after TXA administration, this participant will be replaced in the study. However, data collection for the participant will continue to the trial end.

Data collected from the replaced participants will be used.

## Trial treatment

### Description and regulatory status of investigational medicinal product(s)

TXA is sold globally under a variety of trade names for the treatment of bleeding due to general or local fibrinolysis in adults and children from one year of age ^30^. Several brands are licenced for use globally.

Participants will receive a licenced marketed form of TXA which will be purchased in each country.

### Known drug reactions and interaction with other therapies

TXA solution for injection should not be added to blood for transfusion, or to injections containing penicillin.

### Trial restrictions and the use of concomitant medication

Participants should receive all clinically indicated treatments. There is no restriction on the use of concomitant medication. In the event non-trial TXA is given during the time of the PK blood sampling, the dose, route of administration, date and time should be recorded on the case report form (CRF).

### Assessment of compliance with treatment

IV, IM or oral TXA will be given by investigators at the participating site who will record the date and time of administration and who administered the investigational medicinal product (IMP). For the IM route, the body location of each TXA injection will also be recorded. If the IMP is not fully administered, the reason for this will be recorded in the CRF. The following will not be considered non-compliance with the protocol: where a participant dies before receipt of the IMP or where a clinical or protocol allowed reason is given for non-administration of the IMP.

## Pharmacovigilance

Maternal events which occur as a consequence of the CS, or events which commonly occur in this population independent of exposure to the TXA administration, and those which are study endpoints do not need to be reported as an adverse event. Congenital or genetic abnormalities in neonates do not need to be reported as adverse events as these events are collected routinely. All other events fulfilling the criteria below should be reported. If a participant (both maternal and neonate) develops an adverse event, they should be treated in line with local procedures. In the definition below, participant refers to both maternal and neonate (s).

The flow chart of the safety reporting is shown in Additional file 6 (see *Extended data*) ^49^.

More details on pharmacovigilance are available in Additional file 3 (see *Extended data*) ^49^.

### The type and duration of the follow-up of participants after adverse reactions

Each IM injection site will be monitored as detailed in section “Follow up assessments”. Adverse events in the participant and her baby (ies) will be monitored up to day 7 or until death or discharge whichever is earlier. Any suspected unexpected serious adverse reaction (SUSAR) will need to be reported to the Sponsor irrespective of how long after IMP administration the reaction has occurred. All adverse events will need to be followed up until it has resolved or has reached a stable state.

## Statistics and data analysis

### Sample size calculation

Using PFIM 3.2.1 software ^51^ and based on the population pharmacokinetic parameters determined in a meta-analysis of the different pharmacokinetic studies published in healthy volunteers and data through the IV route in trauma patients ^13, 52^, a sample size of 120 participants will allow estimates (relative standard errors < 30%) of the pharmacokinetic parameters of the intravenous, intramuscular and oral administration of TXA. Optimal maternal blood sampling times were evaluated and are as follows: immediately before TXA administration, and 15, 30 minutes, 1h, 2h, 4h, 8h, 12h and 24h after TXA administration. However, if in exceptional circumstances a sample cannot be taken at the optimal stated time, flexibility in blood sampling is allowed as detailed in the section “TXA administration and timing of biological samples”. The actual sampling time must be recorded.

### Planned recruitment rate

Pregnant women will be enrolled until 120 participants with fully evaluable data are included. Evaluable participants must receive the full dose, not vomit the oral dose within 1 hour of administration, and have at least six post randomisation PK blood samples.

### Statistical analysis plan

All statistical calculations will be performed using STATA, unless otherwise specified. All data will be presented in the form of summaries sorted by treatment group (cohort) and participant ID. Tabular summaries will be presented based on the following grouping: IM, IV, Oral solution and No TXA.

For continuous variables, summary statistics will include sample size, mean, standard deviation, median, minimum, and maximum values. Frequencies and percentages will be calculated for categorical variables.

### Pharmacokinetic analysis

All participants who receive the full dose of TXA and did not vomit the oral dose within the first hour and have at least six PK samples obtained after TXA administration to determine maternal plasma concentrations of TXA will be included in the PK data analysis. The actual blood sampling times will be recorded and used in calculations for PK parameter estimation. 

TXA time-courses will be analysed using the nonlinear mixed effect modelling software program Monolix 2020R1 version ^53^, as previously described ^52, 54– 56^. Briefly, parameters will be estimated by computing the maximum likelihood estimator of the parameters without any approximation of the model (no linearization) using the stochastic approximation expectation maximization (SAEM) algorithm combined to a Markov chain Monte Carlo (MCMC) procedure (to ensure full convergence, the MCMC will be fixed to 20 and the iteration number to 1000). Different error models will be investigated (i.e. multiplicative, proportional and/or additive error models) to describe residual variabilities (expressed as σ, square root of σ ^2^), and the between-subject variabilities (expressed as ω, square root of the variance ω ^2^) will be ascribed to an exponential model. The Bayesian information criterion (BIC) will be used to test different hypotheses regarding the model, i.e.:

i. covariate effect(s) on pharmacokinetic parameter(s)
ii. residual variability model (proportional versus proportional plus additive model)
iii. structure of the variance-covariance matrix for the ω parameters.

Main covariates of interest in the population will be age, bodyweight (BW) and renal function, IV fluid and blood transfusion volume. Parameter estimates will be standardised for a mean standard covariate using an allometric model: P _i_ = P _STD_ x (COV _i_/COV _STD_) ^PWR^ where P _STD_ is the standard value of parameter and P _i_ and COV _i_ are the parameter and covariate values of the i ^th^ individual. The PWR exponents may be estimated from the data. However, for bodyweight, allometric scaling theory dictates that these are typically 0.75 and 1 for clearance and volumes terms, respectively ^46^. The goodness-of-fit of each model will be evaluated by visual inspection of the individual concentration-time courses, the observed-predicted (population and individual) concentration scatter plots and the prediction-corrected visual predictive checks.

**Placenta transfer and neonate heel prick PK levels:** Concentrations will be presented in tabular form with mean, median, standard deviation and range as appropriate. For each time point, comparisons across groups will be done using the analysis of variance. We will adjust for time between drug administration and sampling. Where both an umbilical cord and neonate heel prick sample is available, these will be used to build a PK model with gestational age and birth weight as the main covariates.

### Pharmacodynamic analysis

**D-dimer:** Descriptive statistics for D-dimer concentration in maternal blood will be presented in tabular form with mean, median, standard deviation and range (minimum and maximum) as appropriate. For each time point, comparisons across groups will be done using the analysis of variance. The PK data will be combined, and analyses may be conducted to determine a relationship between exposure and the effect on D-dimers.

### Safety parameters

**Full blood count and renal function parameters:** Descriptive statistics for baseline and follow-up will be presented for each laboratory parameter. Changes from baseline as well as shift tables for laboratory parameters will be presented. All laboratory values will be classified as normal, below normal (low), or above normal (high) based on normal ranges supplied by the central laboratory. Frequencies of abnormal values will be presented in tabular form. For purposes of analyses, laboratory results based upon standardized units will be used.

**Vital signs:** Descriptive statistics for blood pressure, heart, and respiratory rate will be presented in tabular form with mean, median, standard deviation and range (minimum and maximum) as appropriate. Number (%) of women with abnormal values in each randomised group will be presented.

**Expected adverse events (collected routinely as outcomes for all participant):** For each event, the number (%) of women in each randomised group will be presented.

**Other adverse events:** Adverse events will be coded by primary system organ class and preferred term using the Medical Dictionary for Regulatory Activities (MedDRA). The number of events (n) and number (%) of participants with events will be presented. Events will be presented separately for maternal and neonates.

**IM injection skin reaction:** Number (%) of women with a reaction and severity of reaction will be presented.

### Efficacy

**Blood loss at 2 hours**: Descriptive statistics for maternal blood loss at 2 hours post-partum and total blood loss will be presented in tabular form with mean, median, standard deviation and range (minimum and maximum) as appropriate. Comparisons across groups will be done using the analysis of variance.

**Clinical diagnosis of PPH**: Number (%) of women in each randomised group will be presented.

### Interim analysis

There are no planned interim analyses.

### Procedure(s) to account for missing data

If one or more blood samples are not collected, the reason for this will be recorded in the CRF.

Individual missing covariate data will be ignored in the PK model.

## Data management

### Data collection

Information required in the research protocol will be collected first onto a paper CRF and transferred to an electronic case report form (eCRF). The CRF can be viewed at https://ctu.lshtm.ac.uk/woman-ptxa/. Anonymization of the participants will be ensured by using the trial participant’s screening number as their unique ID. This will be recorded on each document needed for the research.

### Data confidentiality

The persons responsible for the quality control of the study will take all necessary precautions to ensure the confidentiality of information relating to the IMP, the study, the study participants and in particular the identity of the participants and the results obtained.

These persons, as well as the site investigators themselves, are bound by professional secrecy.

During and after the clinical study, all data collected about the study participants and sent to LSHTM CTU by the investigators (or sent to other collaborators) will be anonymised.

Under no circumstances will the names and addresses of participants be shown.

The trial will comply with relevant Data Protection regulations including the European Union General Data Protection Regulation.

The Sponsor will ensure that appropriate consent is in place to access any personal information about the participant which is necessary for the quality control of the study.

Additional information on data processing and storage of documents and data is available in Additional file 3 (see *Extended data*) ^49^.

## Monitoring, audit and inspection

### General organisation

The Sponsor (LSHTM) will ensure the safety and respect of individuals who have agreed to participate in the trial. The Sponsor have in place quality assurance systems for monitoring the implementation of the study at the study sites. To contact the Sponsor, see Additional file 7 ( *Extended data*) ^49^.

### Monitoring

The trial will be conducted in accordance with the current approved protocol, International Conference on Harmonisation-Good Clinical Practice (ICH-GCP), each participating country’s relevant regulations and the trial’s written procedures. A monitoring plan will be made based on the risks identified in the risk assessment. The LSHTM Clinical Trials Unit (CTU) or country delegate will monitor the trial to ensure the rights, safety, and wellbeing of the trial participants and to ensure the accuracy of the data. All site investigators will be trained in the trial procedures and have extensive guidance. LSHTM CTU will require investigators and their institutions to provide access to source data and documents and all trial related documents for monitoring, audits, ethics committees review and regulatory inspection. All trial-related and source documents including medical records, original consent forms and original CRFs must be kept safely. Investigators must plan in advance of the trial start where the trial-related documents will be stored and how they will be accessed. All documents must be made available when required for monitoring/audit/inspection during the course of the trial and for up to 10 years after the end of the overall trial.

### Audits/inspections

The Sponsor will also be responsible for auditing all aspects of the trial. The site Principal Investigators (PIs) agree to accept the quality assurance audits carried out by the Sponsor as well as the inspections carried out by relevant RECs and regulatory authorities to ensure adherence to the protocol, GCP and relevant regulations.

## Ethical and regulatory considerations

### Consent

Please see the section “Methods for informing and obtaining consent” for details on methods for informing and obtaining consent from the research participants.

### Legal obligations

***The Sponsor's role.*** This trial is sponsored by the LSHTM and its responsibilities coordinated by the LSHTM CTU. The CTU may delegate responsibilities to third parties which will be outlined in relevant agreements. The responsibilities of the CTU will be overseen by the Trial Management Group. with day to day responsibilities with the Trial Manager.

***Peer review.*** The trial was funded after an open competition with blinded peer review by Wellcome and Bill & Melinda Gates Foundation.

***Financial and other competing interests for the Chief Investigator, Principal Investigators at each site and committee members for the overall trial management.*** The personnel involved in this trial has no financial or other competing interests to disclose. PIs will be compensated for their time needed to oversee the trial conduct. Research staff will be employed at each site to carry out the trial procedures. An agreement with each site will be in place prior to the start of the trial.

### Insurance

LSHTM accepts responsibility attached to its sponsorship of the trial and, as such, would be responsible for claims for any non-negligent harm suffered by anyone as a result of participating in this trial. The indemnity is renewed on an annual basis and LSHTM assures that it will continue renewal of the indemnity for the duration of this trial.

### Access to the final trial dataset

Each site will have continuous access to their own data as the trial is ongoing. The final dataset will be reviewed and published by group authorship consisting of members of the Protocol Committee and key participating site collaborators.

Once all pre-planned analyses are completed, the totally anonymised dataset, protocol, published manuscript, data dictionary and any other relevant trial documents will be made freely available on the LSHTM CTU data platform.

## Dissemination policy

### Dissemination policy

As Sponsor, LSHTM has the right and responsibility to ensure the results of this study are published. The main publication will be done as a group authorship consisting of the Protocol Committee. Once the pre-specified analysis is completed and data made freely available, anyone can use the trial data.

A final study report will be to be sent to the relevant regulatory authorities and ethics committees within one year of the end of the trial.

There are no plans to notify all participants of the outcome of the trial. However, participants can request a copy of the final results and each site will maintain a log of participants/families who would like a copy. LSHTM CTU provide copies to each site to send onwards.

### Authorship eligibility guidelines and any intended use of professional writers

This study is being conducted as an academic collaboration. All parties who contribute significantly to this study will be named in the final publication.

Professional medical writers will not be hired to write dissemination material about the results of this trial.

## Roles and responsibilities

### Role of trial Sponsor and funder

The funders had no role in the design of the trial and will not have a role in its conduct, data collection, analysis, and interpretation, manuscript preparation, review, and approval, and publication of the results. The Sponsor is responsible for the approval of any substantial amendment which may be needed to the protocol. After approval is given, the Sponsor must obtain, prior to implementing the amendment, approval from the relevant regulatory authorities and ethics committees. The Sponsor is responsible for reporting serious adverse events as per each country’s requirement and all serious breaches to relevant regulatory authority and ethics committees.

### Roles and responsibilities of trial management groups and individuals

**Protocol Development Committee:** This includes the Chief Investigators (CIs), site PIs and participating clinicians and study staff. Its role is to ensure that the study protocol is scientifically appropriate and that all ethical, regulatory and scientific aspects of the trial have been considered. If the protocol requires amending, this committee will review and recommend any changes. The final decision for any amendment to the protocol resides with the Sponsor.

Members of the Protocol Development Committee:

•    Haleema Shakur-Still: Study design, drafting and finalising the protocol

•    Ian Roberts: Study design, drafting and finalising the protocol

•    Rizwana Chaudhri: Study design

•    Stanislas Grassin-Delyle: Pharmacokinetic methods and analysis

•    Monica Arribas: Study design and drafting the protocol

**Data Monitoring Committee (DMC):** The primary responsibility for monitoring and final decisions about safety of participants in the trial lies with the Sponsor. Independent oversight of the safety of participants will be provided by an independent DMC. The composition of the DMC is provided in Additional file 8 (see *Extended data*) ^49^.

The DMC will review on a regular basis accumulating safety data (adverse events and injection site reactions) from the ongoing trial, and advise CIs regarding the continuing safety of current participants and those yet to be recruited. Data on the type, frequency and severity of adverse events in mother and neonate will be reported to the DMC.

The DMC membership includes expertise in clinical trials, the use of TXA in obstetrics and gynaecology, care of women during childbirth and postnatal period and care of the newborn. The committee members are familiar with the safety profile of the drug.

The DMC Charter includes, but is not limited to, defining:

• the schedule and format of the DMC meetings;
• the format for presentation of data;
• the method and timing of providing interim reports.

The DMC members are independent of the Sponsor, ethics committees, regulatory agencies, investigators, clinical care of the trial participants, and all trial operations.

**Trial Management Group:** The Trial Management Group will comprise the CI, trial manager, data manager and statistical expert. They will have responsibility for the day to day management of the trial. They will meet regularly to ensure that the trial is progressing according to the protocol.

### PI’s responsibilities

Coordination within each participating hospital will be through a site PI whose responsibility will be detailed in an agreement in advance of starting the trial and will include:

• personally supervise the study at site;
• before and if needed during the trial, obtain all institutional appropriate approvals / favourable opinion;
• delegate trial related responsibilities only to suitably trained and qualified personnel;
• document delegation of duties to appropriately qualified persons;
• train relevant medical, nursing and other staff to ensure that they remain aware of the state of the current knowledge, the trial and its procedures;
• agree to comply with the final trial Protocol and any relevant amendments;
• ensure that all potentially eligible participants are considered promptly for the trial;
• ensure consent is obtained in line with approved procedures;
• ensure that the data are collected, completed and transmitted to the CTU in a timely manner;
• ensure all adverse events are reported promptly to the CTU;
• ensure blood samples are collected and prepared in line with the protocol and trial guidance;
• ensure the investigator site file is up-to-date and complete;
• account for trial drug at their site;
• ensure appropriate storage of trial drug;
• ensure the trial is conducted in accordance with ICH-GCP and relevant country-specific regulations including clinical trial regulations and data protection laws;
• allow access to source data, including participants’ medical records for monitoring, audit and inspection;
• be responsible for archiving all original trial documents including medical records, investigator’s study file, consent forms and data forms for at 10 years after the end of the trial.

## Ethics approval and consent to participate

The protocol was approved by the London School of Hygiene and Tropical Medicine’s Ethics Committee (ref: 21255), University of Zambia Biomedical Research Ethics Committee (UNZABREC, Ref: 933–2020), Zambia Medicines Regulatory Authority (ZAMRA, Reference: DMS/7/9/22/CT/102), Zambia National Health Research Authority (NHREB), National Bioethics Committee Pakistan (NBC, Ref:4-87/NBC-532/20/409), the Drug Regulatory Authority of Pakistan [DRAP, Reference: F. No. 16-16/2020 DD (PS)], the Ethical Review Board of MCH Centre-Pakistan Institute of Medical Sciences Hospital (Reference: F1-1/2015/ERB/SZABMU/583) and the Ethics Committee of the Federal Government Polyclinic Hospital (Reference: FGPC.1/12/2020/Ethical Committee) (Additional files 9, 10, 11, 12, 13, 14, 15, 16 in *Extended data*) ^49^.

## Discussion

The WOMAN-PharmacoTXA trial will provide important data on the PK, PD and safety of TXA and the comparison of IV, IM and oral administration in women giving birth by CS. The WOMAN trial, which studied the effect of TXA for the treatment of PPH, showed that every 15 minutes delay in giving TXA reduced the benefit by 10%. If TXA given by IM or oral liquid route achieves therapeutic levels in blood to reduce bleeding, more women are likely to receive the treatment sooner. This trial is the first step toward developing another route of TXA administration to prevent PPH in settings where personnel trained to insert intravenous lines are not available.

## Trial status

The current protocol is version 1.2, dated 3 April 2020 (see original protocol in Additional file 3. *Extended data*) ^49^. The protocol was approved by the London School of Hygiene and Tropical Medicine’s Ethics Committee (ref: 21255), University of Zambia Biomedical Research Ethics Committee (UNZABREC, Ref: 933-2020), Zambia Medicines Regulatory Authority (ZAMRA, Reference: DMS/7/9/22/CT/102), Zambia National Health Research Authority (NHREB), the National Bioethics Committee Pakistan (NBC, Ref:4-87/NBC-532/20/409), the Drug Regulatory Authority of Pakistan [DRAP, Reference: F. No. 16-16/2020 DD (PS)], the Ethical Review Board of MCH Centre-Pakistan Institute of Medical Sciences Hospital (Reference: F1-1/2015/ERB/SZABMU/583) and the Ethics Committee of the Federal Government Polyclinic Hospital (Reference: FGPC.1/12/2020/Ethical Committee) (Additional files 9, 10, 11, 12, 13, 14, 15, 16 in *Extended data*) ^49^. Participant recruitment began on the 18 ^th^ December 2020. End of recruitment is planned for 30 June 2021 or when 120 fully evaluable participants have been recruited. 

## Data availability

### Underlying data

No underlying data are associated with this article.

### Extended data

LSHTM Data Compass: WOMAN-PharmacoTXA Trial: Pharmacokinetics and pharmacodynamics of tranexamic acid in women having caesarean section birth - Extended data. https://doi.org/10.17037/DATA.00002164 ^49^.

This project contains the following extended data:

- File02-WHO_trial_registration_dataset.docx (Additional file 02 - WHO WOMAN-PharmacoTXA trial registration dataset)
- File03-WOMAN_PharmacoTXA_protocol.pdf (Additional file 03 - WOMAN PharmacoTXA tirial protocol)
- File04-PIS_and_consent_form.pdf (Additional file 04 - Patient Information Sheet and Consent form)
- File05-Consent_procedure_overview.pdf (Additional file 05 - Trial overview of the Consent procedure)
- File06-Safety_reporting_flowchart.pdf (Additional file 06 - Trial safety reporting flowchart)
- File07-Main_contacts.pdf (Additional file 07 - Contact details of the Clinical Trials Unit- LSHTM and Sponsor)
- File08-Data Monitoring Committee.pdf (Additional file 08 - Composition of the Data Monitoring Committee)
- File09-LSHTM_Ethics_approval.pdf (Additional file 09 - LSHTM (Sponsor) Ethics Approval Letter)
- File10-Unzabrec_approval.pdf (Additional file 10 - UNZABREC Approval Letter)
- File11-ZAMRA_approval.pdf (Additional file 11 - ZAMRA Approval letter)
- File12_NHREB_approval.pdf (Additional file 12 - Zambia NHREB Approval letter)
- File13-Pakistan_NBC_approval.pdf (Additional file 13 - Pakistan NBC Approval Letter)
- File14-DRAP_approval.pdf (Additional file 14 - DRAP Approval Letter)
- File15-PIMS_Hospital_approval.pdf (Additional file 15 - Pakistan Institute of Medical Sciences Hospital ERB Approval Letter)
- File16-FGPC_Hospital_approval.pdf (Additional file 16 - Federal Government Polyclinic LEC Approval Letter)

### Reporting guidelines

LSHTM Data Compass: SPIRIT guidelines for “WOMAN-PharmacoTXA trial: Study protocol for a randomised controlled trial to assess the pharmacokinetics and pharmacodynamics of intramuscular, intravenous and oral administration of tranexamic acid in women giving birth by caesarean section”. https://doi.org/10.17037/DATA.00002164 ^49^.

Data are available under the terms of the Creative Commons Attribution 4.0 Unported license (CC-BY 4.0).
