# Supplementary material for: WOMAN-PharmacoTXA trial: Study protocol for a randomised controlled trial to assess the pharmacokinetics and pharmacodynamics of intramuscular, intravenous and oral administration of tranexamic acid in women giving birth by caesarean section
Source: Wellcome Open Res. 2021 Jun 16;6:157. [Version 1] doi: 10.12688/wellcomeopenres.16884.1 (PMC8264807; doi:10.12688/wellcomeopenres.16884.1)
Supplement: Peer review history

# Peer review: Reviewer response for version 1

**DOI:** 10.21956/wellcomeopenres.18625.r44508

**Published:** 2021-07-07

**Authors:** David Faraoni

**Affiliations:** 1 Texas Children's Hospital, Houston, TX, USA; 2 Baylor College of Medicine, Houston, TX, USA

In this article, the authors describe the study protocol of a prospective randomized trial that will aim to assess the pharmacokinetics and pharmacodynamics of TXA administered either orally, intravenously, or intramuscular in woman undergoing elective C section. 

 The primary objective and endpoint of the study will be to compare the pharmacokinetics profile of the 3 different routes of administration. 

 The authors will also looked at fibrinolytic inhibition (using D-dimers), neonatal blood levels, and adverse events. 

 The authors are presenting a strong clinical study that will certainly leads to clinical important finding. Finding alternative routes of administration of TXA in bleeding patients has been a priority to help reduce mortality associated with bleeding in low-income country. The present study will certainly be an important contribution to the literature. 

**Two limitations can be identified in this protocol:** 

 1. Although d-dimers have been extensively used to estimate fibrinolytic activation, d-dimers are not specific and can be influenced by several other factors. It would have been interesting for the authors to include other traditional tests to estimate the degree of fibrinolytic activation/inhibition (e.g., t-PA activated ROTEM, plasmin generation, PAP complexes). 

 2. The study is not designed or powered to compare the efficacy and safety of the different routes. However, this study is a crucial initial step to gather data and defined dose regimen before a large randomized controlled trial can be performed.

Is the study design appropriate for the research question?

Partly

Is the rationale for, and objectives of, the study clearly described?

Yes

Are sufficient details of the methods provided to allow replication by others?

Yes

Are the datasets clearly presented in a useable and accessible format?

Yes

Reviewer Expertise:

Hemostasis and thrombosis in pediatric and adult cardiac surgical patients, pharmacokinetics and pharmacodynamics of antifibrinolytics

I confirm that I have read this submission and believe that I have an appropriate level of expertise to confirm that it is of an acceptable scientific standard.

# Peer review: Reviewer response for version 1

**DOI:** 10.21956/wellcomeopenres.18625.r44504

**Published:** 2021-06-28

**Authors:** Homa K. Ahmadzia

**Affiliations:** 1 Division of Maternal-Fetal Medicine, Department of Obstetrics and Gynecology, School of Medicine and Health Sciences, George Washington University, Washington, DC, USA

Arribas *et al. *present a compelling need for determining alternative routes of TXA administration, particularly in low resource settings. An important contribution from their work will be adding information about transplacental TXA transfer and neonatal levels when TXA is giving prior to cord clamp. There are a few additional suggestions or items of clarification that can strengthen the manuscript:

 1) How was the TXA intravenous route administered - IV infusion or bolus? And were the start and/or end times of administration recorded?

 2) Please note on page 4 in the description of the IV TXA group there are a few more recently published studies of TXA PKPD in pregnant women (see citations below, some of which I was involved with ^1^^,^^2^^,^^3^). The first citation listed also included some breast milk concentrations of TXA ^1^.

 3) Were there pre-defined PD targets selected for d-dimer and if so what literature supported those? 

 4) In the three trials mentioned on top of page 5 where TXA was administered prior to cesarean, what was the 'n' of the participants studied?

 5) On page 5 a more recent TRAAP2 study (citation below ^4^) findings are not included for preventative use of the TXA at the time of cesarean.

 6) When was cesarean section time defined? Time of entry into the operating theater or time of skin incision or time of delivery of baby? This would be good to clarify since interpretation of findings and time at sustained PK target given administration prior to delivery would be most relevant with respect to time of skin incision. At least in the US, surgical centers time of entry to room and time of skin incision can be variable (approx. 15 min to 45 min) depending on how long it takes to obtain regional anesthesia.

 7) To clarify further on estimated blood loss this was this clinically estimated by a primary surgeon, research staff member or any provider at the time of delivery?

 8) Given two central labs measured, d-dimer and this measurement can vary by machine/site - was this factored into consideration in the analysis plan?

Is the study design appropriate for the research question?

Yes

Is the rationale for, and objectives of, the study clearly described?

Yes

Are sufficient details of the methods provided to allow replication by others?

Yes

Are the datasets clearly presented in a useable and accessible format?

Not applicable

Reviewer Expertise:

Prevention of postpartum hemorrhage, PKPD studies in pregnancy, tranexamic acid

I confirm that I have read this submission and believe that I have an appropriate level of expertise to confirm that it is of an acceptable scientific standard.

## References

[1] : Optimal use of intravenous tranexamic acid for hemorrhage prevention in pregnant women. *Am J Obstet Gynecol* .2020; 10.1016/j.ajog.2020.11.035 10.1016/j.ajog.2020.11.035 33248975PMC8149481

[2] : Population pharmacokinetics and pharmacodynamics of Tranexamic acid in women undergoing caesarean delivery. *Br J Clin Pharmacol* .2021; 10.1111/bcp.14767 10.1111/bcp.14767 33576009PMC8355246

[3] : Application of a plasmin generation assay to define pharmacodynamic effects of tranexamic acid in women undergoing cesarean delivery. *J Thromb Haemost* .19(1) : 10.1111/jth.15114 221-232 10.1111/jth.15114 33001565PMC7875467

[4] : Tranexamic Acid for the Prevention of Blood Loss after Cesarean Delivery. *New England Journal of Medicine* .2021;384(17) : 10.1056/NEJMoa2028788 1623-1634 10.1056/NEJMoa2028788 33913639
